# Fabrication of Porous Materials by Spark Plasma Sintering: A Review

**DOI:** 10.3390/ma12030541

**Published:** 2019-02-12

**Authors:** Dina V. Dudina, Boris B. Bokhonov, Eugene A. Olevsky

**Affiliations:** 1 Lavrentyev Institute of Hydrodynamics SB RAS, Lavrentyev Ave. 15, 630090 Novosibirsk, Russia; 2 Department of Mechanical Engineering and Technologies, Novosibirsk State Technical University, K. Marx Ave. 20, 630073 Novosibirsk, Russia; 3 Institute of Solid State Chemistry and Mechanochemistry SB RAS, Kutateladze str. 18, 630128 Novosibirsk, Russia; bokhonov@solid.nsc.ru; 4 Department of Natural Sciences, Novosibirsk State University, Pirogova str. 2, 630090 Novosibirsk, Russia; 5 Powder Technology Laboratory, San Diego State University, San Diego, CA 92182, USA; eolevsky@sdsu.edu

**Keywords:** spark plasma sintering, porous materials, pressureless sintering, space holders, reactive sintering, inter-particle contacts

## Abstract

Spark plasma sintering (SPS), a sintering method that uses the action of pulsed direct current and pressure, has received a lot of attention due to its capability of exerting control over the microstructure of the sintered material and flexibility in terms of the heating rate and heating mode. Historically, SPS was developed in search of ways to preserve a fine-grained structure of the sintered material while eliminating porosity and reaching a high relative density. These goals have, therefore, been pursued in the majority of studies on the behavior of materials during SPS. Recently, the potential of SPS for the fabrication of porous materials has been recognized. This article is the first review to focus on the achievements in this area. The major approaches to the formation of porous materials by SPS are described: partial densification of powders (under low pressures, in pressureless sintering processes or at low temperatures), sintering of hollow particles/spheres, sintering of porous particles, and sintering with removable space holders or pore formers. In the case of conductive materials processed by SPS using the first approach, the formation of inter-particle contacts may be associated with local melting and non-conventional mechanisms of mass transfer. Studies of the morphology and microstructure of the inter-particle contacts as well as modeling of the processes occurring at the inter-particle contacts help gain insights into the physics of the initial stage of SPS. For pre-consolidated specimens, an SPS device can be used as a furnace to heat the materials at a high rate, which can also be beneficial for controlling the formation of porous structures. In sintering with space holders, SPS processing allows controlling the structure of the pore walls. In this article, using the literature data and our own research results, we have discussed the formation and structure of porous metals, intermetallics, ceramics, and carbon materials obtained by SPS.

## 1. Introduction

An enormous potential of porous materials for structural and functional applications stimulates the development of the processing methods of these materials [1,2,3,4,5,6]. Processing of porous metals through bubbling gas into a molten metal lacks control over the percentage of porosity, size of pores, and the structure of the pore walls [7]. By powder sintering, porous structures can be produced using removable space holders and pore formers or by allowing only partial densification of the powder by limiting the pressure and/or temperature. When space holders are used, mixtures of the powder of the target material and the space holder powder are prepared. The space holder can be removed after the final sintering step is complete; it can also be removed after cold compaction or low-temperature sintering. After the removal of the space holder, the porous compact may require further sintering at a higher temperature. Sintering of hollow particles or spheres allows obtaining porous materials with a closed-cell structure; the size of particles or spheres determines the pore size in the material.

In order to improve the quality of inter-particle sintering and control the structure of the pore walls, sintering assisted by electric field can be used. For the preparation of porous materials, electric current can be applied in different modes. High-voltage consolidation for the production of porous metals has been presented in a number of publications [8,9,10,11,12,13,14,15]. In a recent article, Minko and Belyavin reviewed the studies of the structure and properties of porous titanium, niobium, and tantalum obtained by high-voltage consolidation [9]. Yurlova et al. [10] used high-voltage consolidation to produce porous compacts from tantalum powders. In their process, the powders were pre-pressed before being subjected to the action of an electric pulse; no pressure was applied during the consolidation stage itself. Mironov et al. [14] sintered hollow steel spheres by allowing a pulse of electric current from a capacitor to pass through the sample. At the same time, the sample was axially compressed by using a flat inductor. Better sintering between the hollow spheres was achieved when a copper coating was deposited on them increasing the electrical conductivity of the surface.

Spark plasma sintering (SPS) has been firmly incorporated into laboratory and industrial practice as a sintering method offering tools to control the microstructure and phase composition of the sintered materials [15,16,17,18,19]. SPS uses the action of pulsed direct current and pressure and was developed in search of ways to preserve a fine-grained structure of the sintered material while eliminating porosity and reaching a high relative density. Recently, a potential of the SPS devices for the fabrication of porous materials has been recognized, the process flexibility in terms of the choice of the heating rate and heating mode being the main advantage over other sintering methods. As a large number of articles have been published in the past few years, a separate review was needed to highlight the successes and formulate unresolved problems in this area. 

**Figure 1:**
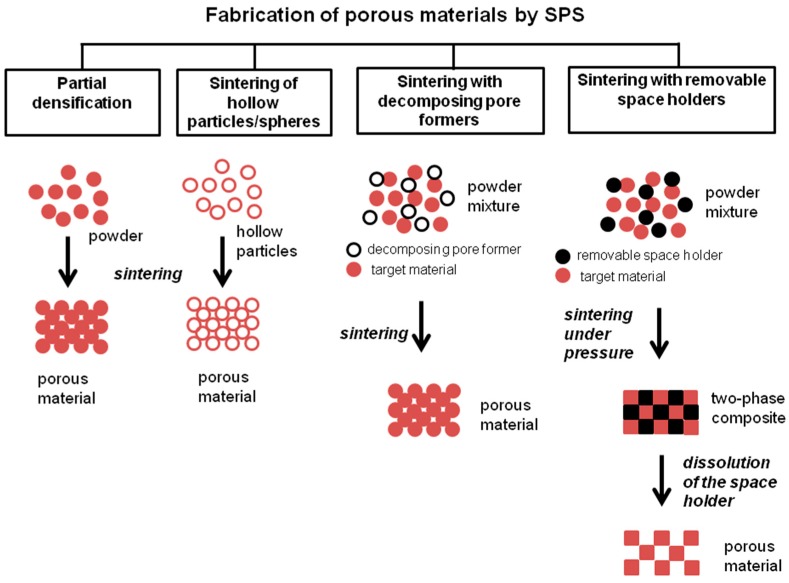
Approaches to the fabrication of porous materials by SPS.

In the present review, we aim to discuss the fabrication approaches, structure formation, and properties of porous materials obtained by SPS. Similar to other sintering methods, with the use of SPS, porous materials can be obtained by allowing partial densification of the material, sintering of hollow or porous particles, sintering with decomposing pore formers, or with space holders to be removed after sintering (Figure 1). Some conventional studies on SPS of various materials include the so-called “densification maps” [15], which can be used as a guide for obtaining materials with controllable final porosity.

**Figure 2:**
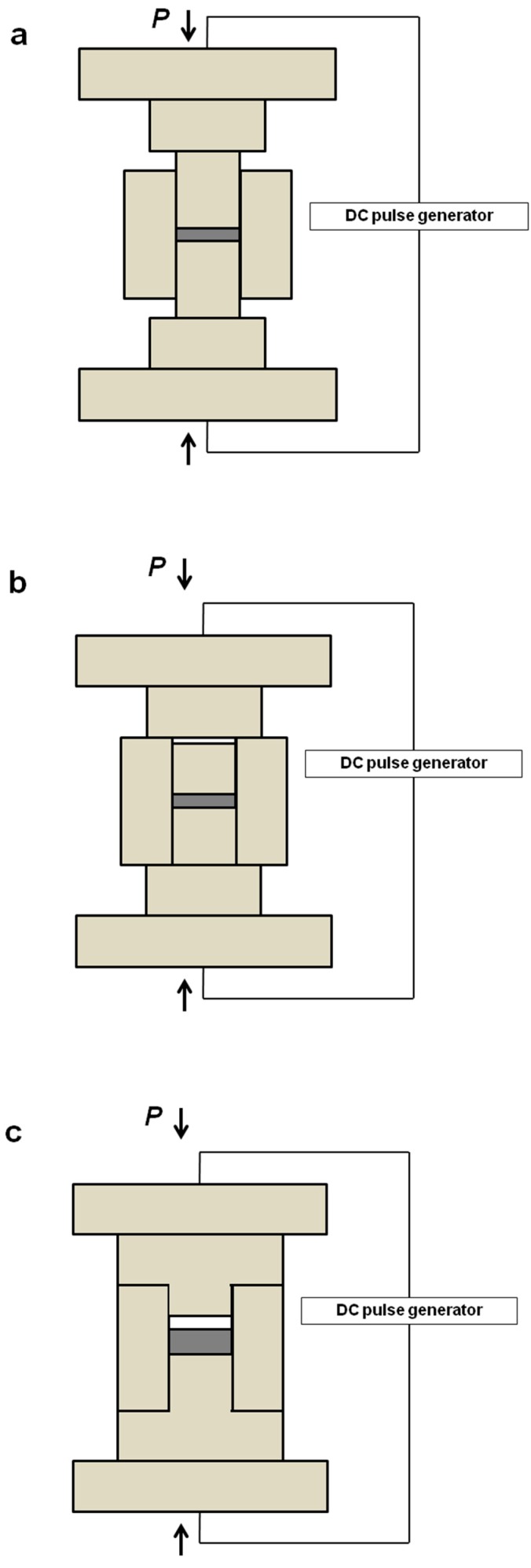
Tooling for pressure-assisted (**a**) and pressureless SPS ((**b**) [20,21,22], (**c**) [23,24]).

Tooling configurations that can be used for the preparation of porous materials are demonstrated in Figure 2a–c. Porous materials can be formed in the commonly used SPS tooling (Figure 2a) in combination with low temperature and/or pressure. Although the sample is not to experience pressure in the pressureless SPS experiments (Figure 2b–c), a certain pressure should be applied to the assembly to enable the passage of current, as no external heaters are present in the SPS chambers. The geometry of the assembly is, therefore, modified, such that the pressure is sustained by the die and not the sample. For that, short punches (Figure 2b) [20,21,22] or T-shape punches (Figure 2c) [23,24] can be used. In the case of short punches, the sum of the height of two punches and that of the sample is smaller than the height of the die. Table 1 provides examples of porous materials obtained by SPS–metals, alloys, intermetallics, ceramics, composites, and carbon materials. 

## 2. Fabrication of Porous Materials by Partial Densification and Sintering of Porous and Hollow Particles/Spheres

Partial densification of powders during SPS has been used in a number of investigations to produce porous metals, alloys, intermetallics, ceramics as well as carbon-based materials (Table 1). Incomplete sintering is ensured when low pressures and/or low temperatures are used. Sintering can be conducted using pre-consolidated compacts [40] or powders freely poured into the die cavity [15,21,47,48,49,50,51]. In this section, we will discuss the development of inter-particle contacts during the formation of porous structures by SPS, sintering of hollow/porous particles and hollow spheres. Studies on the formation of porous intermetallic and ceramic materials via reactive SPS will also be reviewed.

### 2.1. Development of Inter-Particle Contacts During SPS

Physics of inter-particle sintering in electric current-assisted processes is a fundamentally important question, as contacts between the particles are regions of higher resistance due to both geometrical factors and factors related to the altered chemistry of the particle surfaces (e.g., the presence of oxide films on metals). Furthermore, the formation of inter-particle contacts is a key issue in the development of porous materials from powders, as those determine the robustness and strength of the sintered objects. The quality of inter-particle sintering can also influence the functional characteristics of the porous materials (e.g., electrical conductivity, thermal conductivity). Therefore, the mechanisms responsible for the formation of necks during SPS should be studied to enable control over the structure and properties of the porous compacts. 

**Figure 3:**
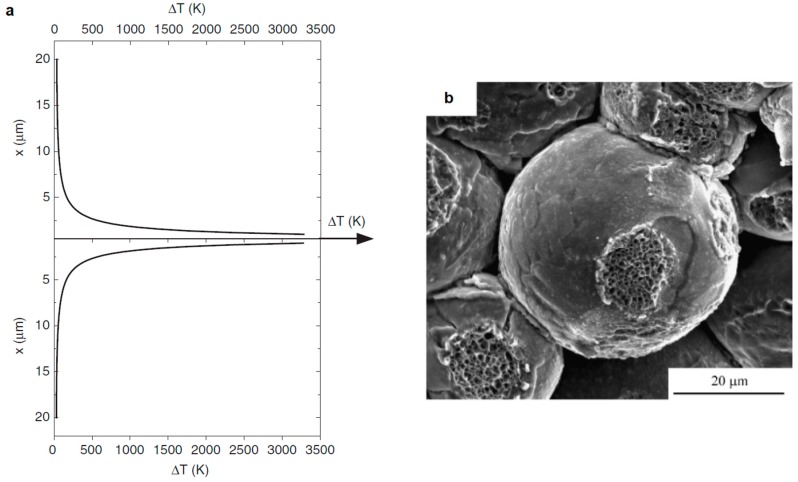
(**a**) Calculated temperature distribution along the line connecting the centers of two copper particles (the current passing through the sample was 1000 A, the sample diameter was 20 mm, the copper particle diameter was 20 μm), (**b**) the morphology of the fractured neck between the copper particles (SPS at 660 °C, 20 MPa, the relative density of the sample was 80.1%). Reprinted from Song et al. [29], Copyright (2005), with permission of John Wiley & Sons.

Song et al. [29] analyzed the temperature distribution in the vicinity of the contact between two spherical particles of copper experiencing the action of pulses of direct current. The following equation for the temperature increase ΔT of a microunit of the material was derived

(1) $$\Delta T=\frac{16}{\pi^{2}}\frac{I_{p}^{2}\rho \Delta t}{\rho_{m}C{\left[r^{2}-{\left(r-x\right)}^{2}\right]}^{2}}$$

where $I_{p}=\frac{4r^{2}}{\varphi^{2}}I_{s}$, ρ is the resistivity of the material, $\rho_{m}$ is the density of the material, C is the heat capacity of the material, $I_{s}$ is the electric current passing through the sample, r is the particle radius, φ is the sample diameter, $I_{p}$ is the electric current passing through the contact, Δt is the total duration of pulses applied in sequence, x is the distance from the point of contact between the particles. The calculations of the temperature distribution at the contact of two copper particles (Figure 3a) allowed the authors to suggest that the inter-particle contacts formed by melting and rapid solidification. Smaller grains than those of the initial copper were observed in the neck area (Figure 3b). However, Ye et al. [79] argue that, in calculations conducted by Song et al., the temperatures are overestimated, as not heat dissipation from the contact area into the particle volume though thermal conduction is taken into account. Theoretical and experimental analysis of the SPS process of TiAl powders detected no overheating of the inter-particle contacts [80]. In the experimental part of this work, metallographic investigations were used in search of local structural changes in the alloy in the vicinity of the inter-particle contacts. It was concluded that, as heat conduction in metallic materials is fast, no significant temperature differences can be stabilized over distances of the order of the particle size (for particles 100 μm or smaller).

**Figure 4:**
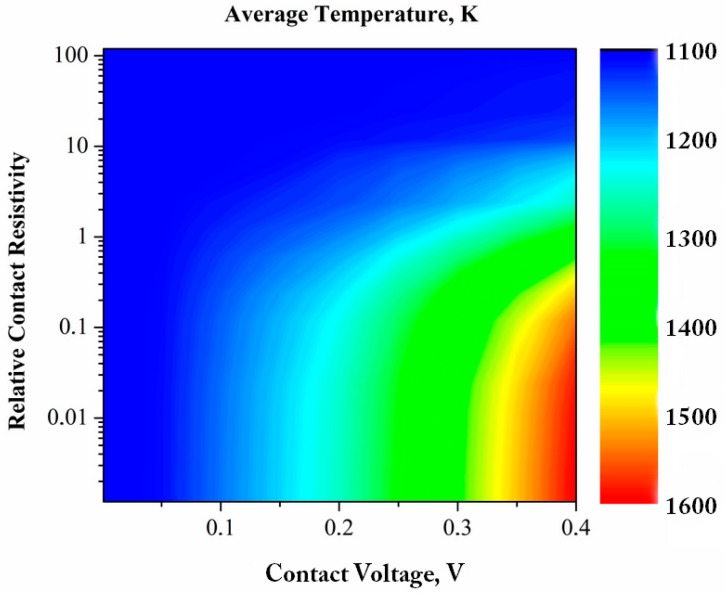
Parametric isolines of the average temperature obtained by modeling of resistance heating of spherical copper particles 10 μm in diameter covered with oxide films. Reprinted from Olevsky & Dudina [15], Copyright (2018), with permission of Springer Nature.

A rigorous analysis of the temperatures and temperature gradients at the contacts between particles covered with oxide films and carrying an electric current was conducted by Kuz’mov et al. [81]. They studied the effect of micro-non-uniform temperature distribution on the rheological behavior of a metal powder with surface-oxidized spherical particles. It was noted that the temperature field at the scale of a particle can be described by a stationary heat-conduction equation, as the time during which the heat is spread over a particle is much shorter than the pulse length or the period of the current. The authors emphasize the role of the oxide films in the temperature distribution over the contact region. Indeed, oxide films are present on particles of many metallic materials and should be taken into account in the analysis of the particle heating. Furthermore, the authors noted that the Branly effect, which is an insulator–conductor transition in a granular medium under the influence of an electromagnetic wave generated by a spark, is absent in pure copper powders in an oxygen-free environment. At the same time, the oxidized powders exhibit an abrupt increase in conductivity under an applied voltage exceeding a certain threshold [82]. The analysis presented in [81] showed that the contact temperature may be substantially different from the average macroscopic temperature of the sample, up to melting of the contact upon a slight increase in the macro-temperature. Figure 4 shows the results of calculations in the form of parametric isolines of the average temperature for the case of resistance heating of spherical copper particles 10 μm in diameter covered with oxide films. The contact voltage is the potential difference between the centers of the particles. The relative contact resistivity $k_{\rho }$ was calculated as

(2) $$k_{\rho }=\frac{h}{r}\cdot \frac{\sigma_{0}}{\sigma },$$

where h is the thickness of the oxide film, r is the particle radius, $\sigma_{0}$ is the electrical conductivity of the material of the particle and σ is the electrical conductivity of the oxide film. Even at low specific voltage drops typical of SPS, the temperature gradient in the contact region between the powder particles with a size of 10 μm and larger exceeds 10^6^ K m^−1^. The contact temperature gradient varies non-monotonically with the thickness of the oxide film, showing a well-pronounced maximum. A higher temperature of the contact region results in higher plasticity of that portion of the particle in comparison with the rest of its volume. Since bridges between particles are regions of major deformation, especially at the early stages of consolidation, the electric current heating establishes conditions favoring faster shrinkage of the material than during traditional furnace heating at the same macro-temperature and external pressure.

**Figure 5:**
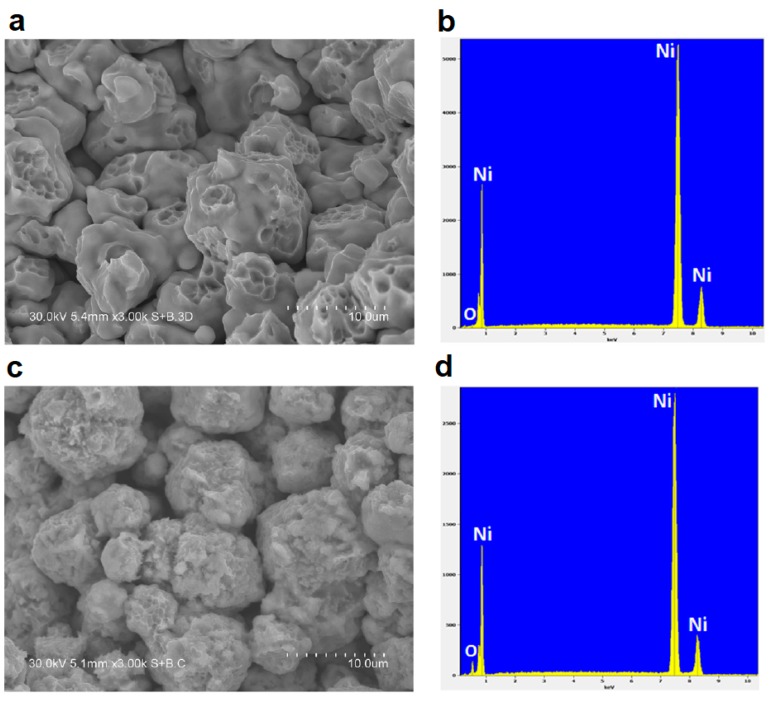
Fracture surface (**a**,**c**) and results of the energy-dispersive spectroscopy analysis (**b**,**d**) of nickel compacts consolidated by SPS from a partially oxidized nickel powder, (**a**,**b**) SPS in contact with graphite foil, (**c**,**d**) SPS in contact with copper foil. Reprinted from Dudina & Bokhonov [25], Copyright (2016), with permission from Elsevier.

Dudina and Bokhonov showed that clean (oxide-free) metallic surfaces are crucial for the neck formation during SPS [25]. Indeed, when a partially oxidized nickel powder was sintered in contact with graphite foil, which supplied carbon that could diffuse along the particle surfaces and reduce nickel oxide NiO, necking was pronounced, the necks showing dimpled fracture surface characteristic of ductile metals (Figure 5a). When copper foil was used instead of graphite foil to contact the sample, NiO remained in the sintered material (on the particle surfaces) hindering the neck formation (Figure 5c). Figure 5b,d shows the results of the energy-dispersive spectroscopy analysis of nickel compacts sintered in contact with graphite and copper foil, respectively: oxygen signals are visible in the spectrum taken from the compact sintered in contact with copper, while they are not detected in the spectrum of the compact sintered in contact with graphite.

**Figure 6:**
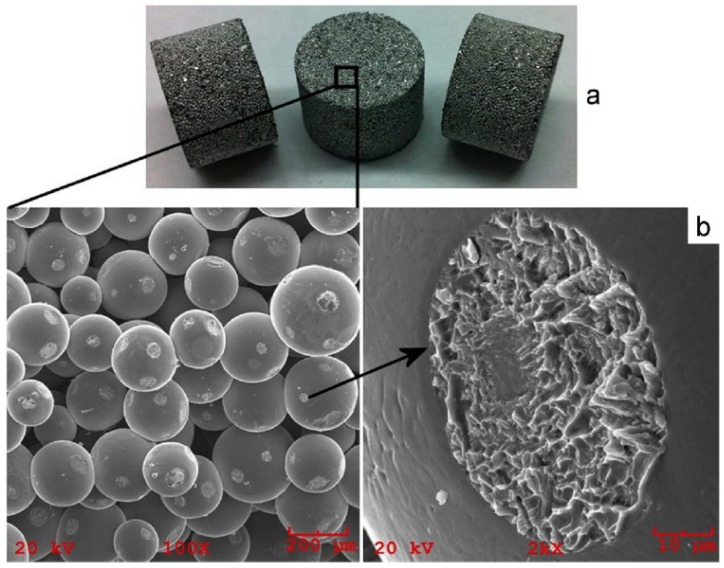
Porous Ti5Al2.5Fe compacts (**a**), contacts between the particles showing dimpled fracture surface (**b**). Reprinted from Yamanoglu et al. [24], Copyright (2016), with permission from Elsevier.

Experiments show that pressureless SPS can result in the formation of both conventional necks [24] and necks with unusual morphologies [26]. Porous Ti5Al2.5Fe alloy compacts were obtained by pressureless SPS using T-shape punches [24]. The Ti5Al2.5Fe powder with spherical particles was subjected to sintering at 750, 800, and 850 °C. A contact pressure was maintained at 5 MPa throughout the process, but was completely supported by the die (Figure 2c). Compacts sintered at 750, 800, and 850 °C showed a porosity of 29.1, 28.7, and 28.4%, respectively. The size ratio of the necks formed between the particles to the particle diameter (equal to 0.3) corresponded to the initial stage in classical sintering. The dimpled fracture surface of the necks indicated enhanced diffusion in these areas during SPS (Figure 6). The inter-particle contacts were quite strong: a porous compact sintered at 850 °C exhibited a compressive strength of 300 MPa.

Aman et al. [26] reported evidence of the unconventional neck formation in copper compacts sintered by pressureless SPS and suggested that the ejection mechanism was operating. In a study by Dudina et al. [20], evidence of melting was found not only in the inter-particle regions when an electrolytic copper powder was processed by pressureless SPS (using an assembly shown in Figure 2b); some ligaments of the porous material had a re-solidified structure. When pressureless SPS experiments for conductive materials are designed in such a way that the sample can carry an electric current but was not pre-pressed before the sintering start, the distribution of electric current across the sample’s cross-section may be rather non-uniform, causing local melting effects (not confined to the inter-particle contacts). In a study by Guyot et al. [83], the contact between spherical copper particles 50 μm in size sintered by SPS at 600 °C and 12 MPa did not show a conventional neck shape. Rather, the contact area was surrounded by sub-micrometric droplet-shaped aggregates, which appear to have been caused by local melting.

**Figure 7:**
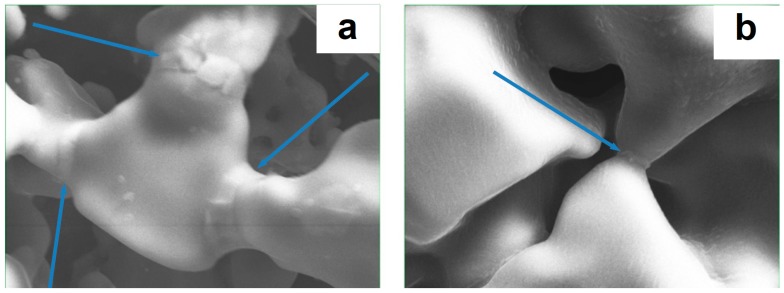
Inter-particle necks formed between V_8_C_7_ particles: (**a**) free pressureless SPS at 1550 °C, holding time 40 min, (**b**) conventional sintering at 1550 °C, holding time 180 min. Reprinted from Giuntini et al. [23], Copyright (2013), with permission from Elsevier.

The effect of the applied electric current on the sintering process can also be traced through the action of high heating rates of the sample by irradiation and conduction from the die heated by the passing electric current. The inter-particle neck growth kinetics during the initial stages of conventional sintering and free pressureless SPS was investigated by Giuntini et al. [23] using a vanadium carbide V_8_C_7_ powder. The maximum temperature in all experiments was 1550 °C. While conventional sintering was not effective in the consolidation process, SPS proved to be successful in terms of the initial stages of densification and inter-particle neck evolution. The difference in the size of the necks formed during free pressureless SPS and conventional sintering is seen in Figure 7a,b. Note the difference in the heating rate in these experiments: in the SPS, the heating rate was 150 °C min^−1^ (from 700 to 1550 °C); in conventional sintering, the heating rate was 10 °C min^−1^.

### 2.2. SPS of Porous Particles

**Figure 8:**
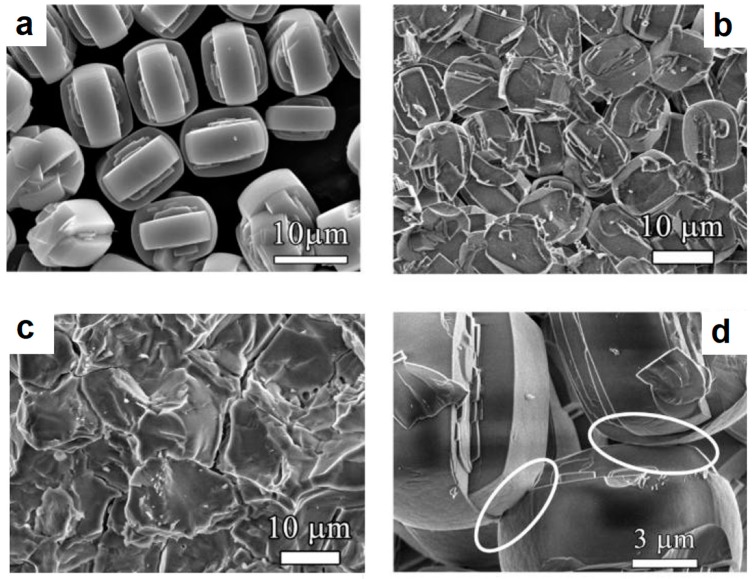
Morphology of silicalite-1 particles (**a**), fracture surface of compacts produces by SPS at 1100 °C (**b**,**d**—different magnifications) and 1300 °C (**c**). Reprinted from Vasiliev et al. [63], Copyright (2010) with permission from the American Chemical Society.

Vasiliev et al. [63] studied the formation of zeolite monoliths by SPS and found that mechanically strong porous materials having hierarchical (multimodal) porosity and containing macro-, meso-, and micropores can be obtained while retaining 85–95% of the surface area of a silicalite-1 powder. After SPS at 1100 °C, structural changes appeared to occur only in the regions of inter-particle contacts (Figure 8a,b,d). Particles of silicalite-1 retained their faceted morphology after SPS at 1100 °C and 20 MPa. During SPS at 1200 °C, the zeolite structure started transforming into α-crystobalite. During SPS at 1300 °C, the structure collapsed, which resulted in a dramatic decrease in the porosity and specific surface area of the material (Figure 8c). The authors attributed the formation of strong inter-particle bonds to local amorphization at the contact points between the zeolite particles; the amorphization process involved the breakage and rearrangement of chemical bonds. One of the advantages of the SPS processing of zeolites is the fabrication of monoliths without adding any non-zeolite binders. It was shown that the strength of the binderless zeolite monoliths produced by SPS was comparable to the strength of monoliths produced by traditional techniques based on the use of binders. 

Similarly, calcined diatomite powders consisting of mainly crystobalite were consolidated into porous compacts by SPS [62]; processing at temperatures of 700–750 °C and a pressure of 16 MPa ensured the formation of relatively strong porous monoliths and preservation of the internal pore structure present in the powder. The porous compacts had bimodal porosity: pores 1–2 μm formed between the powder particles, while smaller pores (about 200 nm in size) were inherited from the pores in the initial powder particles. The consolidation process was restricted to the initial stage such that necking just started. The advantages of the SPS processing were in the high heating rates allowing for a short total temperature exposure and a possibility of applying a compressive stress to the powder assembly. 

**Figure 9:**
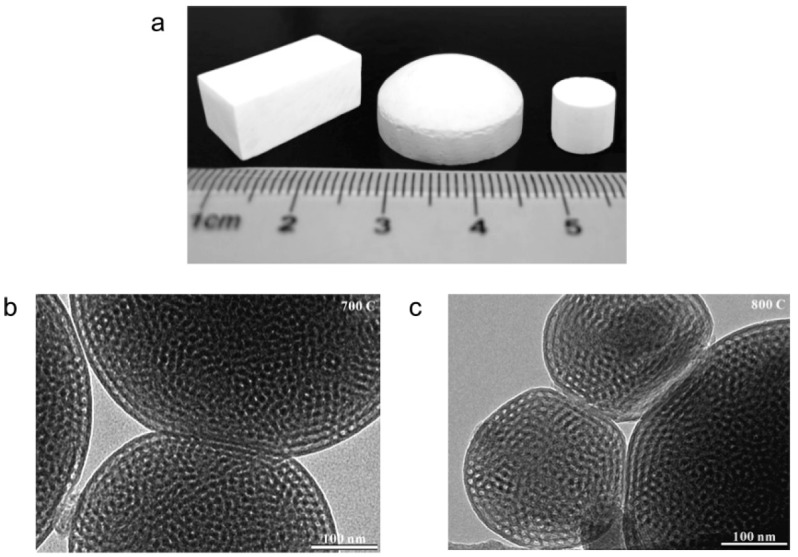
Meso/macroporous silica compacts obtained by SPS at 700 °C and 20 MPa from an amorphous silica powder (**a**), transmission electron microscopy images of the contacts between porous silica particles formed in the compacts subjected to SPS at 700 °C (**b**) and 800 °C (**c**) and a pressure of 20 MPa. Reprinted from Vasiliev et al. [60], Copyright (2006), with permission from the American Chemical Society.

In order to produce silica compacts with bimodal porosity, Vasiliev et al. [60] used the ability of amorphous silica to be viscoelastically deformed above a certain temperature, even below a glass transition temperature. Figure 9a shows a general view of the obtained silica compacts. Transmission electron microscopy investigations showed that the contacting particles of silica were deformed to a certain extent and were ‘fused’ together during SPS at 700 and 800 °C, the deformation degree being greater at a higher temperature (Figure 9b,c). The tensile strength of compacts sintered at 800 °C was 1.6 MPa, which suggests that the partial fusion of mesoporous particles created inter-particle bonding comparable to a gelled and condensed silica network. The partially fused monoliths had a sufficient strength to allow mechanical polishing. 

The successful fabrication of porous bodies containing mesoporosity from mesoporous powders of carbon, silica, and boron nitride has been reported by Dibandjo et al. [61]. Pressureless SPS as well as SPS at 25 MPa was conducted. It was shown that the SPS method makes it possible to maintain the mesopore organization of the solid while producing a macro-sized object from the powder. In comparison with the starting powders, the mesopore volume of the consolidated materials decreased only slightly and was 0.8 cm^3^ g^−1^ for silica, 0.5 cm^3^ g^−1^ for carbon and 0.2 cm^3^ g^−1^ for boron nitride. 

### 2.3. SPS of Core–Shell Particles

Core–shell particles present interesting systems for making porous materials by SPS. Upon heat treatment, core–shell metallic particles transform into their thermodynamically stable alloy counterparts [84]; therefore, the alloy formation occurs in parallel to inter-particle sintering. Depending on the differences between the physical/chemical properties of the shell and the core, the presence of the shell can either hinder [85] or facilitate [86] inter-particle sintering. Bokhonov and Dudina [21] investigated the behavior of the Fe@Pt core–shell particles during pressureless SPS. The Fe@Pt core–shell microparticles were synthesized by galvanic replacement reaction using reduction of H_2_PtCl_6_ aqueous solution by metallic iron. SPS consolidation resulted in the formation of porous compacts, necking between the particles, alloying between the metals, and growth of the crystallites. It was demonstrated that selective dissolution of the core metal (iron) from the SPS-compacts allows producing a porous structure made of Fe(Pt) fcc solid solution. 

### 2.4. SPS of Hollow Particles and Spheres

**Figure 10:**
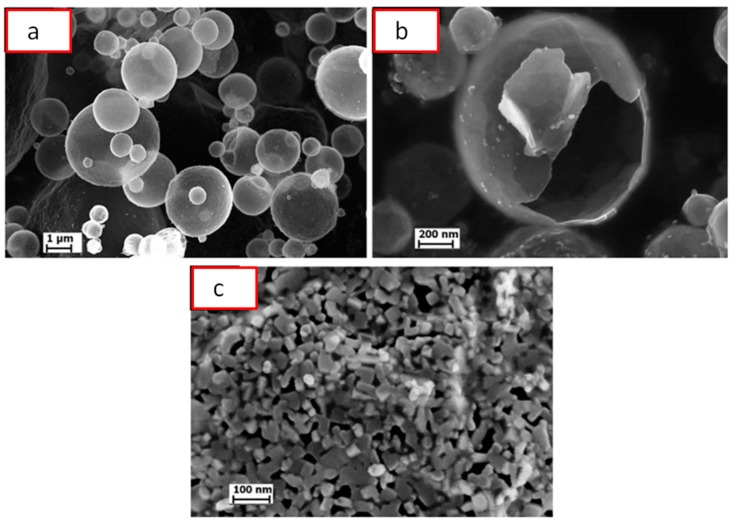
Morphology (**a**,**b**) and wall structure (**c**) of hollow Ni particles obtained by spray solution combustion synthesis. Reprinted from Trusov et al. [22], Copyright (2018), with permission from Elsevier.

**Figure 11:**
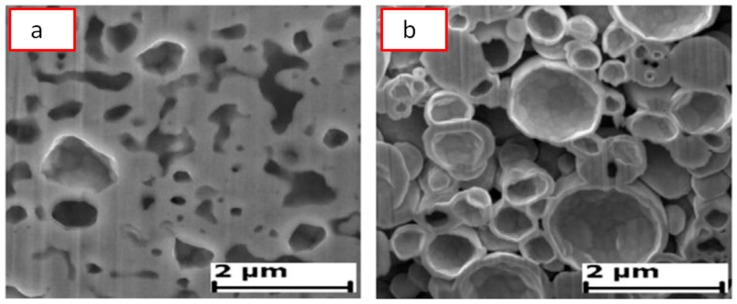
Cross-section of porous nickel obtained by pressureless SPS of hollow Ni particles obtained by spray solution combustion synthesis: (**a**) 500 °C, 30 min, porosity 70%; (**b**) 500 °C, 15 min, porosity 88%. Reprinted from Trusov et al. [22], Copyright (2018), with permission from Elsevier.

SPS of hollow particles allows obtaining porous materials with a closed-cell structure. Trusov et al. [22] studied the structure formation of highly porous nickel during pressureless SPS of hollow Ni particles obtained by the spray solution combustion synthesis. The morphology of the hollow particles and the structure of the walls are shown in Figure 10. SPS at 500 °C for 30 min led to the collapse of the hollow spheres, and a compact with a porosity of 70% formed (Figure 11a). When the holding time was 15 min only, the particle morphology was well preserved leading to the formation of a compact with a porosity of 88% (Figure 11b). According to the authors, the structure of the particle walls, which consisted of Ni nanoparticles and amorphous regions, was of great importance for the sintering behavior of the material. Hollow spheres with walls made of nanoparticles (Figure 10c) showed high sinterability enabled by rapid mass transfer across the particle surface. Samples with a porosity of 93% could withstand 4 MPa in the elastic region in compression. The formation of necks was facilitated and a rigid skeleton was formed from the particles. Noteworthy is the fact that, unlike hollow nickel particles, nickel with a sponge-like structure did not sinter into robust compacts under these conditions. 

The behavior of hollow Pt(Fe) particles during pressureless SPS was investigated by Bokhonov and Dudina [21]. Pt(Fe) hollow particles were obtained by dissolving iron from Fe@Pt core–shell particles in HCl solution. The compositional design of the porous materials was shown to be possible by changing the order of SPS and selective dissolution processing stages. 

Porous materials can also be manufactured from hollow millimeter-sized spheres [55]. In order to make hollow spheres, commercially available polystyrene foam spheres are coated with a metal powder and a binder suspension in a fluidized bed. Then the organic components are removed by heat treatment. Porous materials made by sintering of hollow spheres may show low elastic moduli because of the porosity present in the walls of the spheres and remaining in the walls of the porous compact. Khor et al. [55] suggested using SPS to consolidate hollow spheres into a porous structure and densify the cell walls at the same time. They noted that the porosity in the cell wall cannot be eliminated by conventional vacuum furnace treatment, as temperatures required to induce pore elimination in the cell walls are so high that the porous structure tends to collapse or the material melts. Hence, a method was needed to remove the porosity from the cell walls without damaging the porous structure itself. In order to densify the cell walls, pre-consolidated compacts made of spheres were placed into a graphite die of a SPS apparatus with an yttria-stablized zirconia powder around the sample, the role of which was to sustain pressure applied to the punches and prevent the sample from collapsing under an applied pressure. 

**Figure 12:**
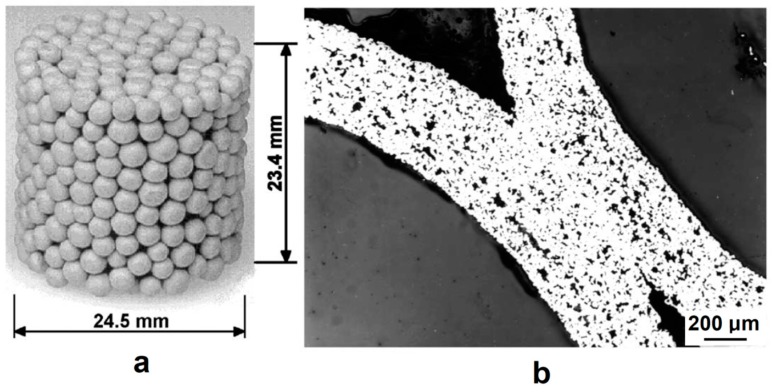
General view of the randomly packed steel sphere sample (**a**), porous structure of the cell wall (**b**). Reprinted from Khor et al. [55], Copyright (2003), with permission from Elsevier.

**Figure 13:**
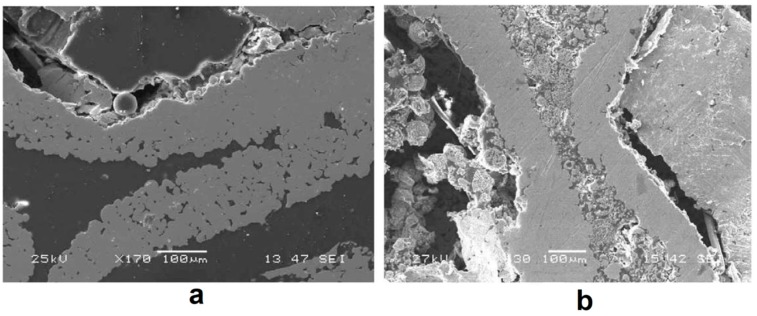
Structure of the cell walls of the steel sphere samples after treatment by SPS at 1000 °C (**a**) and 1200 °C (**b**). Reprinted from Khor et al. [55], Copyright (2003), with permission from Elsevier.

**Figure 14:**
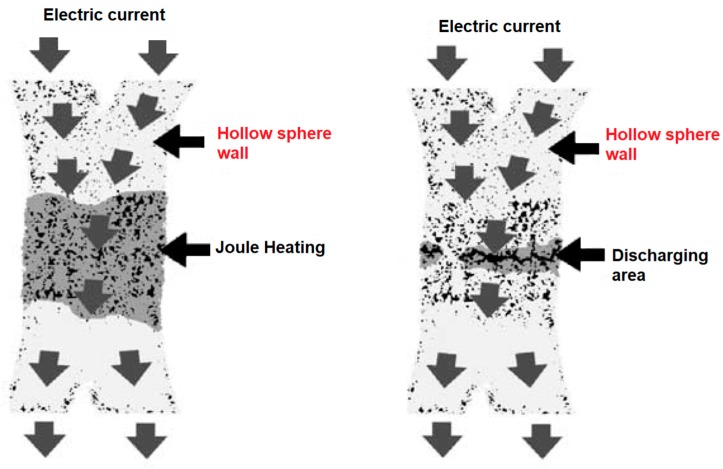
Densification of the wall of steel spheres by SPS. Reprinted from Khor et al. [55], Copyright (2003), with permission from Elsevier.

Figure 12 shows a general view of the randomly packed steel sphere sample and the structure of the cell wall before the SPS treatment. After the treatment, the walls became denser, as can be seen from Figure 13; the densification effect was more pronounced at a higher sintering temperature. The Young’s modulus of the wall was determined using a microhardness tester and was found to increase from about 10 GPa (before SPS) to about 50 GPa (after SPS at 1200 °C). It was suggested that the electric current passing through the walls helps not only eliminate pores but also heal the gaps (defects) in the wall (Figure 14). So, the selected processing was beneficial for retaining the porosity and densifying the pore wall.

### 2.5. Pressureless SPS of Pre-Compacted Porous Compacts

**Figure 15:**
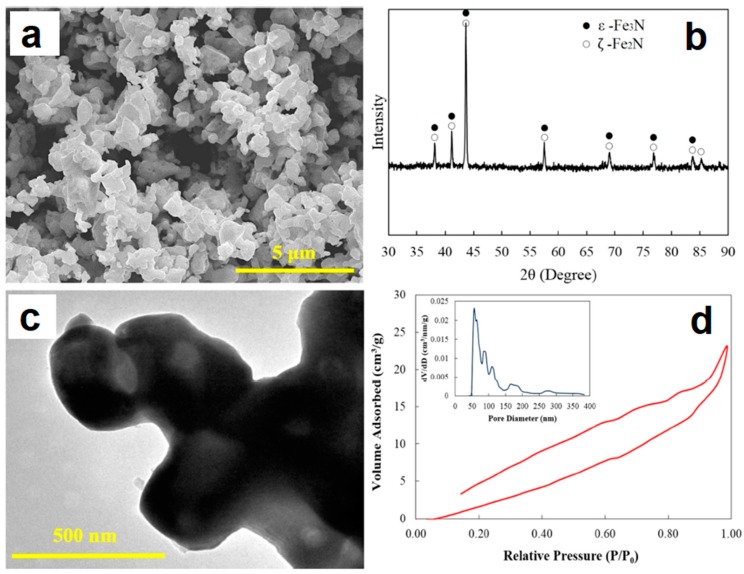
Morphology (**a**), X-ray diffraction (XRD) pattern (**b**), structure in the transmission electron microscope (**c**) and adsorption–desorption isotherm/pore size distribution (**d**) of the iron nitride-based powder. Reprinted from Cui et al. [40], Copyright (2016) by the authors; this article is an open access article distributed under the terms and conditions of the Creative Commons Attribution (CC-BY) license (http://creativecommons.org/licenses/by/4.0/).

**Figure 16:**
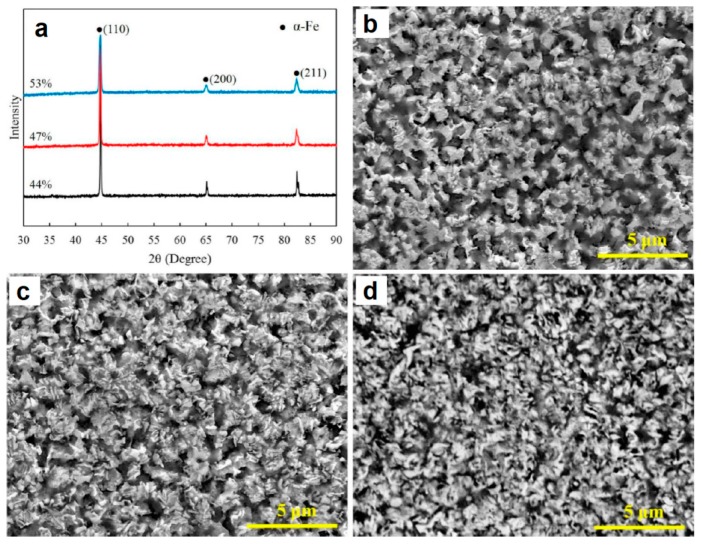
XRD patterns (**a**) and structure of porous iron (**b**–**d**) prepared by pressureless SPS from the pre-compacted iron nitride powder ((**b**) porosity 44%, (**c**) 47%, and (**d**) 53%). Reprinted from Cui et al. [40], Copyright (2016) by the authors; this article is an open access article distributed under the terms and conditions of the Creative Commons Attribution (CC-BY) license (http://creativecommons.org/licenses/by/4.0/).

**Figure 17:**
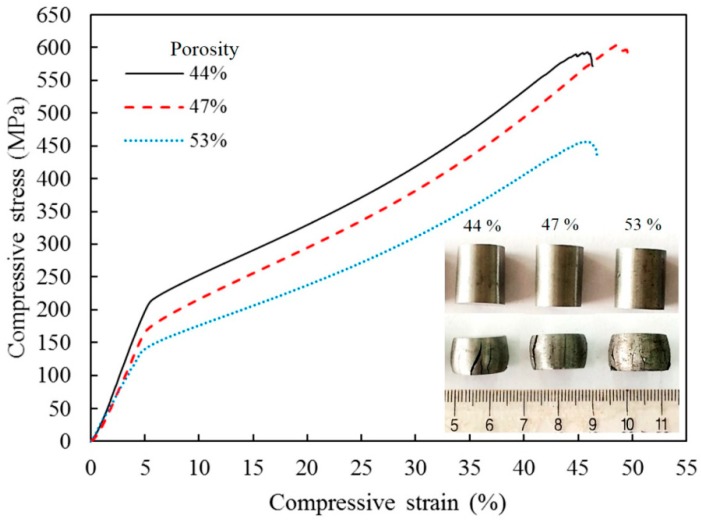
Room temperature uniaxial compressive stress–strain curves of porous iron prepared by pressureless SPS from the pre-compacted iron nitride powder. Reprinted from Cui et al. [40], Copyright (2016) by the authors; this article is an open access article distributed under the terms and conditions of the Creative Commons Attribution (CC-BY) license (http://creativecommons.org/licenses/by/4.0/).

Cui et al. [40] studied the formation of porous α-Fe from pre-compacted iron nitride hollow particles obtained by ammonia treatment of iron oxide powder. Figure 15 provides information on the morphology (a) and phase composition (b) of the powder as well as its fine structure (c) and porous structure (d). The powder consists of iron nitrides. It was compacted at room temperature using different pressures; the compacts were further sintered in a SPS die without applying pressure. The porosity remained in the compact thanks to pressureless sintering (conducted using T-shape punches (Figure 2c) at a relatively low temperature (750 °C) for a short time (the duration of the isothermal holding stage was 5 min). In addition, the compacts gained porosity owing to decomposition of iron nitrides and the formation of α-Fe (Figure 16a). As the compaction pressure of the iron nitride powder was increased from 20 to 60 MPa, the porosity of iron decreased from 53 to 44%. The porous structure of compacts with different porosities is shown in Figure 16b–d. The mechanical properties of porous iron were examined by uniaxial compressive tests at room temperature (Figure 17). These curves are characterized by two regions. In the first linear portion, the compressive stress increases rapidly with increasing strain until the yield point appears at a strain of about 4%. After the yield point, the stress increases slowly in response to the increase in strain indicating a deformation strengthening process.

The possibility of fast fabrication of porous compacts from powders is especially important when one deals with metastable phases. Xie et al. [43] obtained porous Zr_55_Cu_30_Al_10_Ni_5_ metallic glass compacts by SPS of gas-atomized amorphous powders completely avoiding crystallization of the alloy. A compact with a porosity of 33.5% was formed by SPS at 20 MPa and 613 K. This temperature is lower than the glass transition temperature of the alloy. No crystallization took place at the inter-particle contacts or in the particle volume. The sintered porous glassy specimens exhibited a larger plastic strain and a lower Young’s modulus compared with those of the as-cast alloy. An increase in the plastic strain was attributed to the presence of pores in the sintered alloy promoting the generation of multiple shear deformation events.

### 2.6. Reactive Sintering

Reactive pressureless SPS is a promising method to produce highly porous intermetallic and ceramic parts from a mixture of powder reactants in a single technological step. Dudina et al. [51] studied the formation of porous FeAl materials by pressureless reactive SPS. Mixtures of iron and aluminum powders were used as the starting materials. The mixtures of the Fe-40 at %Al composition in the form of loose powders were poured into the die and subjected to pressureless SPS. The sintering conditions were varied to determine the influence of electric current and the heating rate on the formation of FeAl compacts with high open porosities (41–51%). It was found that rapid heating of a loosely packed Fe-Al mixture in the SPS chamber with or without an electric current passing directly through the sample ensures a fast transformation of the reaction mixture into a highly porous FeAl product. It was concluded that the passage of electric current through the sample is not responsible for the preservation of high open porosities. The transverse rupture strength of the FeAl-based compacts produced at 700–900 °C by pressureless SPS ranged between 53 and 72 MPa [50].

**Figure 18:**
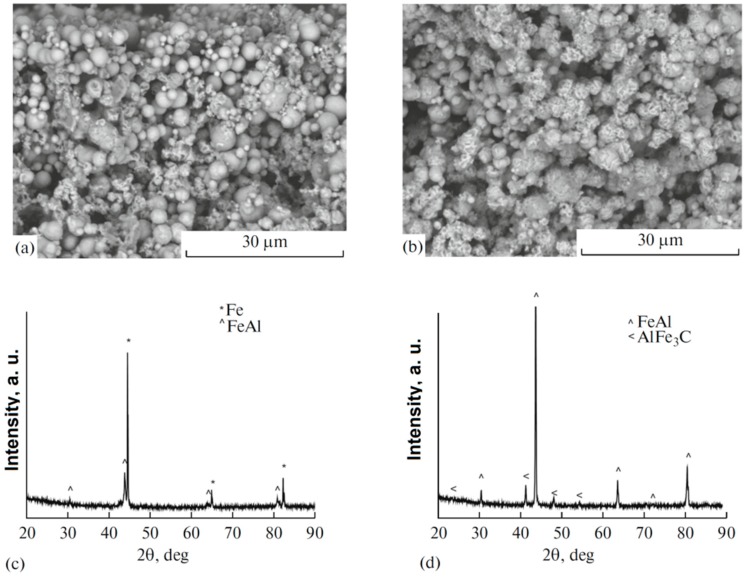
Fracture surface of the compact formed from a mixture of Fe and Al powders by pressureless SPS at 650 °C for 3 min, the top of the sample was not contacting the tooling: (**a**) in the vicinity of the top of the sample, (**b**) in the vicinity of the bottom; XRD patterns taken from the top (**c**) and bottom (**d**) of the sample. AlFe_3_C is an admixture phase formed due to the presence of carbon in the starting iron powder. Reprinted from Dudina [48], Copyright (2017), with permission of Springer Nature.

When the upper flat end of the sample is not in contact with the tooling and the SPS processing times are short, compacts with gradients in the phase composition and structure can form in the synthesized porous materials [48]. As seen in Figure 18, the top of the compact obtained from a mixture of Fe and Al powders contained unreacted iron, while the bottom of the compact contained the product phase only–FeAl.

Simonenko et al. [68] combined the carbothermal synthesis of silicon carbide from finely-dispersed starting SiO_2_–C (obtained by means of the sol–gel technique) with the formation of silicon carbide ceramics during SPS. The goal of their work was to develop a fabrication method of porous silicon carbide ceramics to be used as inert supports for catalysts and for making filters for hot gases and molten salts. SPS was conducted at moderate pressures (22.6–25.5 MPa) in the 1300–1800 °C temperature range. The compacts had porosities in the 42–55% range. The reaction between SiO_2_ and carbon started at 1300 °C. After SPS of the product of the sol-gel synthesis at 1800 °C for 10 min, the synthesis of SiC was still incomplete, the estimated yield of the reaction being only 64%.

**Figure 19:**
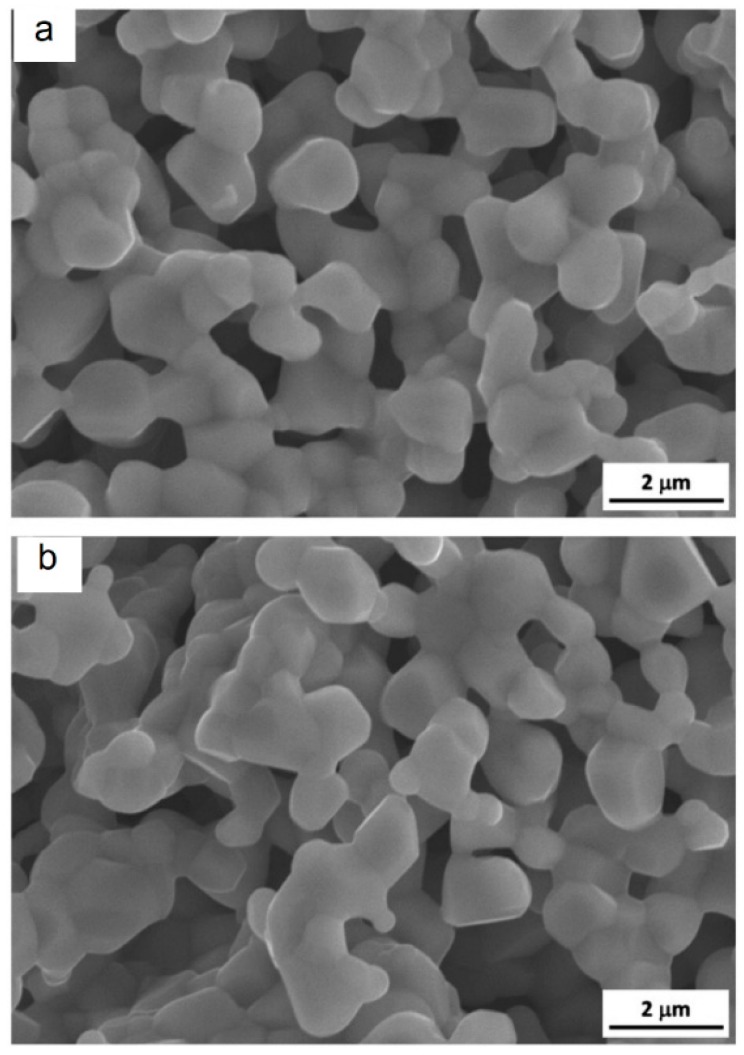
Surface of ZrB_2_ porous compacts synthesized at 1600 °C, holding time 10 min (**a**), 20 min (**b**). Reprinted from Yuan et al. [66], Copyright (2012), with permission from Elsevier.

Reactive synthesis was shown to be suitable for making porous ZrB_2_ ceramics [66]. ZrB_2_ compacts were obtained from ZrO_2_ and B_4_C reactants, the reaction between which is thermodynamically possible at temperatures above 1265 °C. The porosity of the sintered ZrB_2_ compacts was about 70% (Figure 19).

Porous activated carbon-SiC nanowire composite materials were obtained by pressureless SPS processing of activated carbon and silicon mixtures [67]. Pressureless SPS conducted at high heating rates produced SiC nanowires with greater aspect ratios and smoother morphology as compared with products of a conventional low-heating rate synthesis.

### 2.7. Low-Temperature SPS

**Figure 20:**
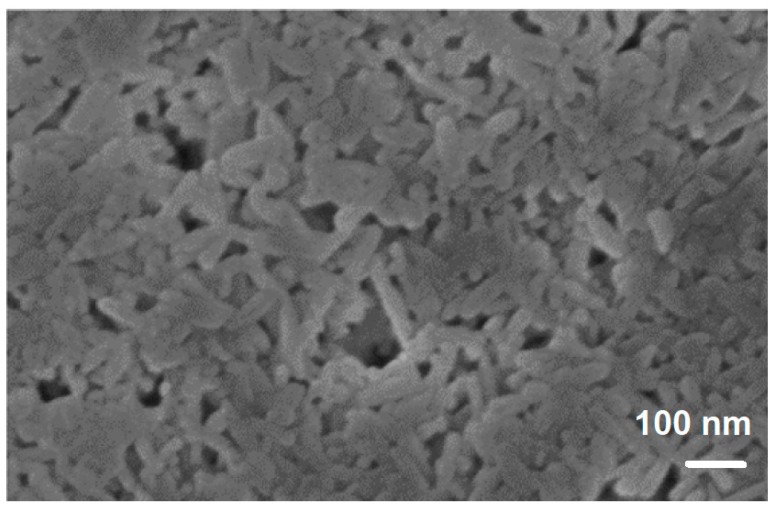
Surface of the partially densified hydroxyapatite sample perpendicular to the direction of applied load, SPS temperature 150 °C. Reprinted from Ortali et al. [58], Copyright (2017), with permission from Elsevier.

Ortali et al. [58] synthesized an amorphous hydroxyapatite precursor at 37 °C and obtained a partially densified compact (with a relative density of 71%) from it by SPS at a record low temperature of 150 °C and an applied pressure of 80 MPa. Biaxial flexural strength of the partially densified samples was 18 ± 5 MPa, which indicates efficient sintering. Low-temperature SPS of the amorphous powder led to crystallization of the apatite. During sintering, the grains changed their morphology: large spherical particles of the initial powder transformed into small needlelike grains (Figure 20). The grains grew preferentially in the plane perpendicular to the direction of the applied load during SPS.

## 3. Fabrication of Porous Materials by SPS Using Space Holders Removed after Sintering or Decomposing During Sintering

In this section, the fabrication of porous materials by SPS using space holders removed after sintering (e.g., by selective dissolution or burning) or space holders decomposing during sintering will be reviewed. Examples of the influence of the removal process of the structure of the target phase and the interaction between the target material and the space holder during sintering will be provided. 

### 3.1. Sintering with Space Holders Removable after Sintering

When the precursor of a porous structure is a two-phase system, the space occupied by the space holder is the future pore space. The sacrificial phase should be fully removable from the sintered compact, which is ensured at concentrations of the space holder exceeding a certain limit depending on the relative particle size (the target phase versus the space holder phase) and morphology. A very popular space holder material is a powder of sodium chloride (NaCl). It can be easily removed by dissolution in water; however, when it is used, the range of the allowed sintering temperatures is not very wide, as the sintering temperature should be lower than the melting point of NaCl. If further heat treatment is necessary, it can be performed after the space holder has been removed. Using NaCl as a space holder, aluminum [7,34,35], titanium [33], and titanium alloys [52] have been fabricated.

Hakamada et al. [34] used aluminum powders with three different sizes—3, 20, and 300 µm—to form aluminum scaffolds with pores ranging from 850 to 1000 µm. For that, NaCl particles 850–1000 µm in size were selected as a space holder material. The pores formed after the NaCl space holder had been dissolved from the Spark Plasma Sintered compacts. The volume content of NaCl in the mixtures with Al was 78%. The size of the pores was pre-determined by the size of the NaCl particles. When small aluminum particles were used, the formation of the pore walls was enabled by particle rearrangement, and the aluminum particles could easily fill the space between the NaCl particles. Filling the space was more difficult when large aluminum particles were used, as plastic deformation of the material was required. It was found that, when the pore walls were formed by 3- and 20-µm particles, i.e., aluminum particles much smaller than those of the space holder, the fabricated porous materials had a higher flow stress than that of the porous material formed from 300-µm aluminum particles.

**Figure 21:**
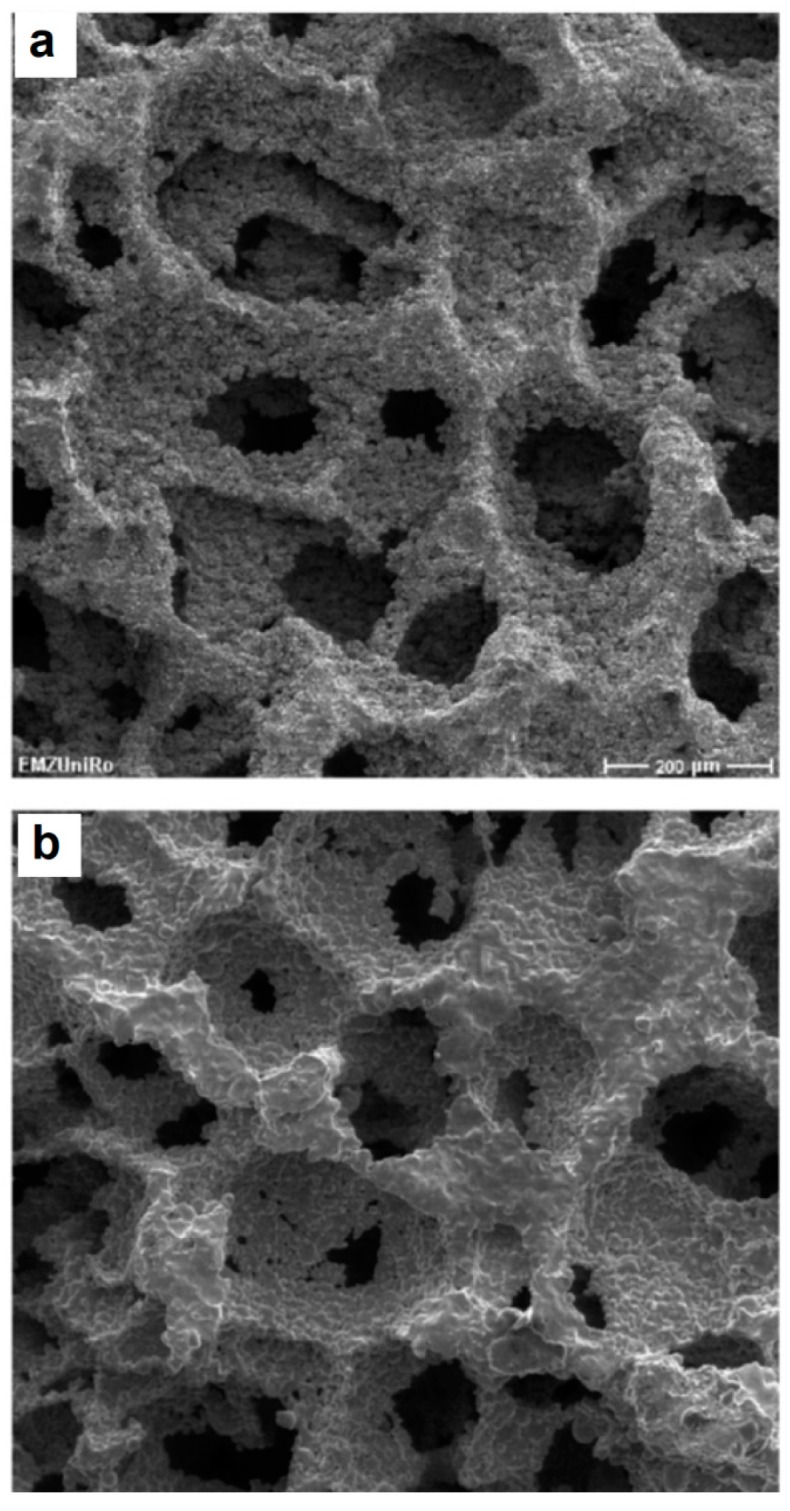
Porous structure of Ti6Al4V obtained by SPS with NaCl as a space holder: SPS at 700 °C, 50 MPa and removal of NaCl (**a**), after heat treatment of the porous compact at 1100 °C by pressureless SPS (**b**). Reprinted from Quan et al. [52]. Copyright (2012), with permission from Elsevier.

The fabrication of porous Ti6Al4V was reported by Quan et al. [52], who used pressure-assisted SPS to produce Ti6Al4V-NaCl compacts and pressureless SPS (conducted at a higher temperature than the first sintering operation) for heat-treating the porous compacts obtained after NaCl had been dissolved (Figure 21). Heat-treatment of the porous compacts resulted in the elimination of small pores and helped densify the walls of the porous structure.

The influence of the applied pressure on the structure of pore walls has been demonstrated by Hakamada et al. [34]. Porous Al sintered with NaCl space holders was tested in compression and a structural study of the deformed specimens was conducted. The cell walls of the porous Al specimen fabricated at 20 MPa bent without fracturing, while those of the specimen sintered 8 MPa underwent brittle fracture. No voids could be seen in the cell walls of the compact sintered at 20 MPa, while some voids were observed in the cell walls of the compact sintered at 8 MPa. These voids indicate insufficient connection between Al particles at a lower sintering pressure.

Zhang et al. fabricated Ti-hydroxyapatite porous compacts with interconnected porosity using SPS and the space holder method [72]. The obtained porous composites contained 5–30 wt % of hydroxyapatite and satisfied the property requirements to implant materials—a low elastic modulus and a sufficient compressive strength. The sintering process was described as influenced by both direct passage of electric current through the sample and heat transfer from the graphite die, the former dominating at low hydroxyapatite contents. It was found that excessive concentrations of hydroxyapatite (greater than 30 wt %) are detrimental for sintering of the titanium scaffold. NH_4_HCO_3_ was used as a space holder and was added in a constant concentration of 5 wt % to the Ti-hydroxyapatite mixtures with different Ti/hydroxyapatite ratios. NH_4_HCO_3_ decomposed into gaseous products, which were eliminated at the early sintering stages and did not influence the composition of the final product. The fabricated porous material contained large pores (of the order of 400 µm) formed in places of the decomposed space holder particles as well as small pores in the walls of large pores formed due to incomplete sintering between titanium and hydroxyapatite and their chemical interaction resulting in the formation of several new phases. It should be noted that, although NH_4_HCO_3_ decomposed at temperatures close to 60 °C, the pores formed in places of NH_4_HCO_3_ particles did not collapse up to 1200 °C—the final sintering temperature. The preservation of pores was possibly due to the presence of hydroxyapatite nanoparticles, which hindered the plastic flow of titanium as well as rearrangement of the titanium particles. The same role could have been played by the products of chemical reactions between titanium and hydroxyapatite as well products of decomposition of the latter.

Space holders are usually used for making porous metals, as it is difficult to find a suitable space holder for ceramic materials. The difficulty lies in the fact that the space holder should be able to withstand temperatures, at which sintering of ceramic particles will occur, and should be easily removable after sintering. However, in some case, such a space holder can be found. An interesting method of producing porous alumina was suggested by Shin et al. [68], who used graphite as a space holder during SPS. After sintering, the graphite was burnt out to produce porous alumina. A similar route was used by Papynov et al. [71] for the production of porous wollastonite; a carbonaceous template was used during SPS and burnt out after sintering.

### 3.2. Removal of the Sacrificial Phase: Effect on the Target Phase

Using the space holder approach, nanoporous silver was obtained from Ag-Fe and Ag-Ni nanocomposites formed by high-energy ball milling of the powder mixtures followed by SPS and selective dissolution of iron or nickel in HCl acid solution [36]. In these cases, iron and nickel served as space holders for the formation of porous silver. Ag-Fe and Ag-Ni are immiscible systems and, during ball milling, mixing between the metals occurred at the crystallite size level resulting in the formation of nanocomposite structures. In these systems, the space holder phases not only occupied the pore space but also participated in recrystallization of the target material through a chemical reaction upon HCl treatment. It was found that silver—the target phase—interacts with HCl solution, which results in the formation of AgCl. Precipitation of silver from AgCl occurs upon its interaction with iron or nickel. So, in parallel to experiencing dissolution by directly reacting with HCl solution, these metals participated in the galvanic replacement reaction

2AgCl + Me = 2Ag + MeCl_2_ (3)

where Me is Fe or Ni. This reaction offers a path for recrystallization of silver governed by the dissolution–precipitation mechanism.

### 3.3. Interaction between the Space Holder and the Target Material

The space holder phase may be reactive towards the target phase. During sintering of the powder mixtures, partial transformation of the space holder material and the target material into compounds or solid solutions can occur. The products of interaction will differ from the two-phase consolidated mixture by their response to leaching treatment. Dissolution of a component from an alloy represents a process of dealloying; in this process, pores form as the structure of the depleted alloy experiences atomic rearrangement. As reported by Mandal et al. [39], SPS of mixtures of copper and zinc powders results in the formation of compacts, in which alloying proceeded only at the inter-particle contacts due to fast processing. The initial mixture of copper and zinc powders was milled under low-energy conditions in order to produce a uniform mixture and not to allow alloying to take place. SPS was selected as a fast sintering method to eliminate the problem of zinc evaporation. The products of alloying formed a network-like structure within the spark plasma sintered compact. It was possible to control the extent of alloying by changing the sintering temperature. After sintering at 500 °C, the compositional variations from point to point within the material were still significant: Cu-rich and Zn-rich regions could be detected indicating a low degree of the alloy formation between the metals. Soaking the material in HCl solution led to complete dissolution of Zn. Samples sintered at higher temperatures retained zinc after treatment in the same corrosive medium. Selective dissolution of Zn from Cu-5wt %Zn composites consolidated by SPS resulted in the formation of porous copper with a porosity of 40% and pores in the range of 1.5–40 µm.

**Figure 22:**
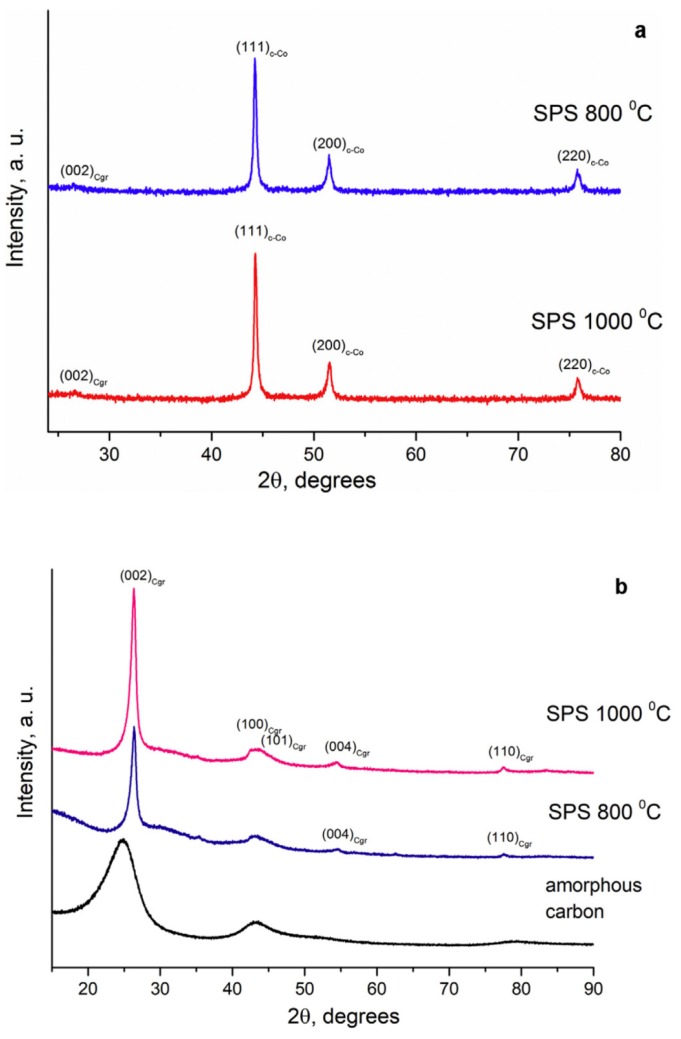
XRD patterns of compacts obtained by SPS of the ball-milled Co-17 wt %C mixture at 800 and 1000 °C (**a**), XRD patterns of the starting amorphous carbon (carbon black) and porous graphitized materials obtained by selective dissolution of cobalt from the sintered compacts (**b**). Reprinted from Bokhonov et al. [78], Copyright (2018), with permission from Elsevier.

**Figure 23:**
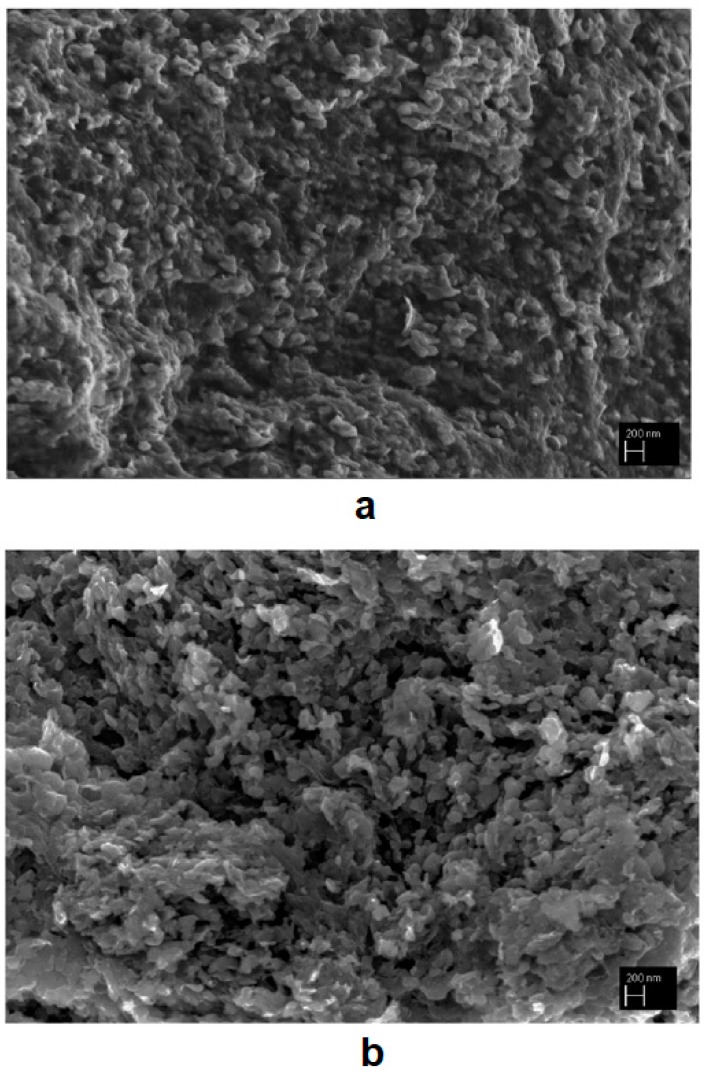
Fracture surface of the compact obtained by SPS of the ball-milled Co-17 wt %C mixture at 800 °C (**a**) and the porous graphitized material obtained by selective dissolution of cobalt from the sintered compact (**b**). Reprinted from Bokhonov et al. [78], Copyright (2018), with permission from Elsevier.

The space holder phase can be selected such that it will influence the crystalline structure of the target material. This influence can be used to advantage if, for example, the space holder phase is a catalyst of the phase transformation in the target phase. This approach was suggested by Bokhonov et al. and initially used to produce porous graphitic materials from amorphous carbon by SPS using nickel as a space holder and a graphitization catalyst [75]. Later, this approach was also tested for iron [77] and cobalt [78] space holders. The mixtures were first subjected to mixing or high-energy ball milling and then consolidated by SPS under a pressure of 40 MPa. In the case of iron, the formation of the Fe_3_C phase complicated the process; upon annealing of the sintered compacts, graphitization was enhanced in samples containing higher concentrations of free (metallic) iron. No nickel or cobalt carbides were observed after SPS of the corresponding mixtures. X-ray diffraction (XRD) patterns of compacts obtained by SPS of the ball-milled Co-17 wt %C mixture at 800 and 1000 °C are shown in Figure 22a. In the presence of cobalt, it is quite difficult to conclude on the graphitization degree of carbon from the XRD data because of the low intensity of the graphite reflections relative to those of cobalt. After cobalt had been dissolved in HCl solution, it became possible to observe the influence of the sintering temperature on the graphitization degree of carbon. The XRD patterns of the porous graphitized materials obtained by selective dissolution of cobalt from the sintered compacts show that the graphitization degree of carbon increased with increasing sintering temperature (Figure 22b). The XRD pattern of the starting amorphous carbon powder allows a comparison to be made between the initial state of carbon and that of the porous carbon after ball milling with cobalt/SPS/selective dissolution treatment. Figure 23 shows the fracture surface of the compact obtained by SPS of the ball-milled Co-17 wt %C mixture at 800 °C and the porous graphitized material obtained by selective dissolution of cobalt from this compact. It can be seen that the graphitic skeleton is made of graphite platelets with a size of 200 nm in their plane and a thickness of 20 nm. The specific surface area of the porous graphitic material is 100 m^2^ g^−1^, which is greater than the specific surface of the amorphous carbon used as a starting material [75].

## 4. Challenges Associated with Processing of Porous Materials by SPS

The fact that the application of electric current and the application of pressure in the SPS are related should be taken into account when pressureless SPS is conducted on samples of loose powders poured into the die cavity without a pre-pressing operation. Under these conditions, pressureless sintering with electric current passing through the sample can lead to the formation of regions in the sample, in which the temperatures are higher than that of the rest of the sample. The temperature can rise in regions experiencing high current densities in the case of non-uniform current distribution in the sample. In the sintered materials, such ‘hot spots’ can differ in the phase composition (for reacting powder mixtures) and microstructure from the rest of the sample. For example, when the packing uniformity of a Fe-Al powder mixture was disturbed, areas with an increased reaction yield formed in the Fe-Al porous compacts obtained by pressureless SPS of loosely packed Fe + Al powders [87]. These areas were distinguishable by the naked eye by their altered (darker) color compared with the rest of the sample.

The challenge in making porous structures from powders is preserving porosity and, in some cases, the structure of the particles, while achieving mechanical integrity of the material and a reasonable level of mechanical strength. Therefore, a comparative analysis of the properties of the porous materials obtained by different methods and having the same porosity and pore size should be performed. Further research is needed to understand the influence of the electric current-related processing conditions on the mechanical properties of porous sintered materials. Comparative studies of the properties of materials obtained by pressureless SPS and SPS with space holders and having the same levels of porosity should be conducted to show the advantages of one of these two processing routes. At present, such studies are lacking.

## 5. Potential Applications of Porous Materials Obtained by SPS

From a scientific point of view, the formation of porous materials directly by SPS or via the formation of two-phase composites, from which the space holders are further removed, presents interesting cases for studying the fundamental nature of the influence of high heating rates, electric field, electric current, and temperature gradients on the structure of materials processed by SPS. The fabrication of porous materials by SPS can be controlled in a variety of ways and thus is also attractive from a technological perspective. 

Porous aluminum is a promising energy-absorption material [34,35], while porous Ti-based alloys are designed to become biocompatible materials [24,44]. Porous metallic glass materials are also considered as promising biomaterials due to high corrosion and wear resistance of metallic glass [42]. 

Macro-porous graphitic materials can serve as substrates for holding nano-sized carbon objects [88]. Porous monoliths obtained by SPS from diatomite powders are recommended for water purification and waste water treatment [62]. Partial fusion of silica particles during SPS was suggested as a promising fabrication approach to tailoring the bimodal pore structure and rapid production of mechanically stable monoliths for making membranes, sensors, and catalyst supports [60]. 

SPS was shown to be a suitable method for making porous fuel cells designed using a single-component concept [56]: Sm-doped CeO_2_–Na_2_CO_3_ was as an ionic material and a mixture of metal oxides Li_2x_Zn_1−x_O–NiO–SrO–CuO was as electronic conductor. An interesting idea has been put forward by Ning et al. [57], who used porosity as a structural parameter to tune the properties of a thermoelectric material. In their work, Sb-doped Mg_2_Si_0.5_Sn_0.5_ was prepared using pressureless SPS. Compared with dense samples, those with a porosity of 37% showed a significantly lower thermal conductivity and a higher Seebeck coefficient.

## 6. Conclusions

Processing of porous materials by SPS is currently receiving increased attention. In the past few years, substantial research data have been accumulated on the fabrication of porous metals, alloys, intermetallics, carbon materials, ceramics, and composites by SPS. The following major approaches to the formation of porous materials in the SPS-assisted processing are used: partial densification (pressureless sintering, sintering at low pressures, and/or low temperatures), sintering of hollow and porous particles, sintering of hollow spheres, and sintering with removable space holders. The so-called ‘densification maps’ obtained during conventional studies of SPS can be used as a guide for obtaining materials with controllable final porosity.

Experimental studies and modeling of the processes occurring at the inter-particle contacts offer opportunities to gain new insights into the field effects involved in the SPS.

It was shown experimentally that, during pressureless SPS of conductive materials, the formation of inter-particle contacts can be associated with non-conventional mass transfer mechanisms. 

Carbon of the graphite tooling contacting the specimens helps eliminate oxide films present on the surface of the powder particles, which promotes the formation of metallic contacts. 

The effect of the applied electric current on the sintering process can also be seen through the action of high heating rates. For pre-consolidated specimens, SPS can be used for heating at high rates, which can be beneficial for controlling the formation of a porous structure and morphology of the products of chemical reactions, if those are involved. 

SPS with space holders and processing of hollow spheres by SPS allow controlling the microstructure of the pore walls during the formation of porous compacts.

Currently, SPS of porous materials is a rapidly developing area with a high potential for making energy-absorption materials, bioimplants, high-temperature filters, fuel cells, and thermoelectric materials. 

## Figures and Tables

**Table 1:** Examples of porous materials obtained by SPS.

Porous Material	Production Method	References
Metals	Partial densification during SPS (powders of conventional morphologies)	[20,25,26,27,28,29,30]
	SPS with additives decomposing during sintering	[31,32]
	SPS under pressure with space holders removed after sintering	[33,34,35,36,37,38,39]
	SPS of hollow particles	[22]
	SPS of hollow particles of the material experiencing decomposition upon sintering	[40]
Alloys and intermetallics	Partial densification during SPS (powders of conventional morphologies)	[24,41,42,43,44,45]
	SPS with additives decomposing during sintering	[46]
	Chemical reaction and partial densification during SPS	[47,48,49,50,51]
	SPS under pressure with space holders removed after sintering	[52,53]
	SPS of reaction mixture under pressure with a space holder followed by post-treatment to conduct the synthesis	[54]
	SPS of hollow particles/spheres	[21,55]
Ceramic materials and composites	Partial densification during SPS (powders of conventional morphologies)	[26,56,57,58,59]
	SPS of porous particles	[60,61,62,63,64,65]
	Chemical reaction and partial densification during SPS	[66,67,68,69]
	SPS with a space holder	[70,71]
	SPS with additives decomposing during sintering	[72]
Carbon materials	Partial densification during SPS	[73,74]
	SPS under pressure with space holders removed after sintering	[75,76,77,78]
